# Linking Staff Cases in a Hospital COVID-19 Outbreak Using Electronic Tracking Data

**DOI:** 10.1017/ash.2021.91

**Published:** 2021-07-29

**Authors:** Pragya Dhaubhadel, Margie Pace, Trina Augustine, Seth Hostetler, Mark Shelly

## Abstract

**Background:** Significant outbreaks of SARS-CoV-2 infections have occurred in healthcare personnel (HCP). We used an electronic tracking system (ETS) as a tool to link staff cases of COVID-19 in place and time during a COVID-19 outbreak in a community hospital. **Methods:** We identified SARS-CoV-2 infection cases through surveillance, case investigation and contact tracing, and voluntary testing. For those wearing ETS badges (Centrak), data were reviewed for places occupied by the personnel during their incubation and infectious windows. Contacts beyond 15 minutes in the same location were considered close contacts. **Results:** Over 6 weeks (August 10–September 14, 2020), 35 HCPs tested positive for SARS-CoV-2 by NAAT testing. In total, 18 nurses and aides were clustered on 1 hospital unit, 7 cases occurred among respiratory therapists that visited that unit, and 10 occurred in other departments. Overall, 17 individuals wore ETS badges as part of hand hygiene monitoring. ETS data established potential transmission opportunities in 17 instances, all but 2 before symptom onset or positive test result. Contacts were most often (10 of 17) in common work areas (nursing stations), with a median time of 45 minutes (IQR, 21–137). Contacts occurred within and between departments. A few COVID-19 patients were cared for in this location at the time of the outbreak. However, we did not detect HCP-to-patient nor patient-to-HCP transmission. **Conclusions:** Significant HCP-to-HCP transmission occurred during this outbreak based on ETS location. These events often occurred in shared work areas such as the nursing station in addition to break areas noted in other reports. ETS systems, installed for other purposes, can serve to reinforce standard epidemiology.

**Funding:** No

**Disclosures:** None

